# Barriers and facilitators associated with the use of mental health services among immigrant students in high-income countries: A systematic scoping review

**DOI:** 10.1371/journal.pone.0287162

**Published:** 2023-06-29

**Authors:** Christelle Dombou, Olumuyiwa Omonaiye, Sarah Fraser, Jude Mary Cénat, Karine Fournier, Sanni Yaya

**Affiliations:** 1 Interdisciplinary School of Health Sciences, University of Ottawa, Ottawa, Ontario, Canada; 2 Centre for Quality and Patient Safety Research, Institute for Health Transformation, Deakin University, Melbourne Campus, Burwood, Australia; 3 Centre for Quality and Patient Safety Research, Eastern Health Partnership, Box Hill, Victoria, Australia; 4 School of Psychology, University of Ottawa, Ottawa, Ontario, Canada; 5 Health Sciences Library, University of Ottawa, Ottawa, Ontario, Canada; 6 School of International Development and Global Studies, University of Ottawa, Ottawa, Ontario, Canada; 7 The George Institute for Global Health, Imperial College London, London, United Kingdom; Monash University, AUSTRALIA

## Abstract

**Background:**

Immigrant students face various challenges in high-income countries that can contribute to the decline of their mental well-being upon arrival in their host country. Despite the growing population of these students in several high-income countries, there is inadequate attention given to their mental health needs and their access to mental health services. Thus, this systematic scoping review aimed to identify gaps in existing research relating to the barriers and facilitators associated with access to and use of mental health services in high-income countries.

**Methods:**

Following the PRISMA-ScR checklist as guidance we systematically searched Ovid Medline, APA PsycInfo, Education Source, CINAHL, Web of Science databases for peer reviewed articles related to barriers and facilitators of mental health service use among immigrant students. We conducted a narrative evidence synthesis to highlight barriers and facilitators to the use of mental health services.

**Results:**

Out of the 2407 articles initially found, 47 studies met the inclusion criteria and were considered for this review. The increasing attention towards the mental health concerns of immigrant students and their access to mental health services is evident. However, various barriers like stigma, insufficient knowledge, or adherence to traditional gender roles (such as masculinity) hinder their utilization of these services. On the other hand, factors such as being a woman, having a strong sense of cultural adaptation, or possessing adequate mental health literacy serve as facilitators for accessing mental health services.

**Conclusion:**

These students have unique experiences, and their needs are often unmet. To improve their mental health and use of mental health services, it is important to consider the barriers they face and their unique experience in their specific life context and to develop tailored prevention and intervention programs.

## Introduction

Around the world in 2019, approximately 1 in every 8 individuals, or 970 million people, were reported to be living with a mental disorder, with anxiety and depressive disorders being the most prevalent [1]. Due to the COVID-19 pandemic in 2020, the number of people affected by anxiety and depressive disorders saw a significant rise. Within just one year, estimates indicate an increase of 26% and 28% respectively for anxiety and major depressive disorders [1].

The mental health condition of university students globally can vary depending on several factors such as cultural differences, academic pressures, social isolation, financial difficulties, and access to resources [2]. However, studies have shown that anxiety and depression are among the most common mental health conditions experienced by university students worldwide [3]. Up to 35% of university students worldwide may suffer from a mental health disorder, with depression and anxiety being the most prevalent [3].

The United Nations Migration Agency, International Organization for Migration (IOM) [4], defines an immigrant as "any person who is moving or has moved across an international border or within a State away from his/her habitual place of residence, regardless of the person’s legal status; whether the movement is voluntary or involuntary; what the causes for the movement are; or what the length of the stay is." This definition includes refugees, asylum seekers, migrant workers, international students, and other individuals who have left their country of origin to settle in a new country [4].

As of 2020, the United Nations estimated the number of migrants worldwide to be about 280.6 million people, a number that continues to increase due to globalization [5]. Upon arrival in host countries, immigrants generally have better health status, including mental health, than natives. This phenomenon is known as the "healthy immigrant effect" [6–9]. However, this health status tends to decline over time due to the changes, stress, adaptation, hardships, and unexpected events associated with the migration process [10, 11]. These factors put the mental health of immigrants at risk of deterioration. Although mental health services are available for immigrants in high-income countries, but accessibility may vary due to language barriers, cultural differences, and legal status [12]. Some countries offer specialized mental health services for immigrants, but barriers to accessing these services need to be addressed [12].

### Case of immigrant/international students: Statistics and particular problems

Despite numerous studies on immigration and mental health [13–16], there is a lack of research on the mental health of immigrant students, despite their increasing presence in educational institutions worldwide. According to UNESCO, there were over 5.3 million international higher education students in 2017 [17], with the US alone hosting more than 1 million international students in the 2019/2020 academic year, according to the Institutes of International Education (IIE) [18]. The UK had almost 556,625 international students during the 2019/2020 academic year [19, 20].

Canada had about 530,540 international students in 2020, representing a 135% increase in 10 years, according to the Canadian Bureau for International Education (CBIE) [21]. In Australia, over one in four tertiary students were international students in 2018 [22], with about 686,104 international students in 2020 and 483,484 in February 2021 despite the COVID-19 pandemic [23]. China welcomed almost half a million international students in 2018 and has sent approximately 5,857,100 students abroad since its "reform and opening up" policy in 1978 [24, 25].

### Specific problems faced by immigrant students in host countries

Similar to other immigrants, immigrant students face challenges such as lack of social support, unemployment, high school costs, poverty, that can lead to the deterioration of their mental health upon arrival in their host country. International students encounter additional challenges related to adapting to a new culture, educational system, and language, making them more susceptible to mental health issues [26]. These challenges can affect various aspects of their experience, including socio-cultural, academic, administrative, financial, and personal, at different stages of their journey [27].

According to Bérubé and colleagues, the primary barriers that international students face are culture shock, difficulties in building a social network, adaptation to teaching and learning, language barriers, and racism and prejudice [28]. Culture shock arises when international students have to learn or understand several new elements in a short period to adapt to their new culture, which can lead to isolation, acculturation stress, sleep and eating disorders [28]. Building a social network with local peers is vital to facilitate the integration of international students into their host country’s culture and keep them motivated [26, 29, 30]. However, finding such support can be challenging, and although buddy programs exist, it is not always easy to build strong social ties and integrate into the host country’s community. Zhang and Brunton’s study on Chinese international students in New Zealand highlighted the difficulty of finding social support [31].

Also, international students face challenges in adapting to new teaching methods, assessment modalities, and technological tools, as well as understanding teachers’ expectations, pedagogical approaches, and note-taking methods [32]. Racism and prejudice also amplify mental health deterioration, with approximately 25% of international students in Canada reporting racial discrimination and 29% reporting cultural or religious discrimination [28].

Furthermore, international students face administrative challenges that increase their vulnerability to mental illness. They don’t receive government-funded integration services like permanent residents [33], and must pay exorbitant tuition fees, often triple that of citizens of the host countries. This financial burden can lead to anxiety and other mental illnesses [2]. International students are also under intense pressure to excel in competitive university programs while navigating study permit restrictions. Cultural pressures can cause students to fear speaking up about their difficulties [34, 35], and a lack of understanding of the healthcare system can be another barrier to accessing care.

### Present study

While there has been some research addressing barriers to health services for immigrants [13–16], the literature remains limited and less clear on the general predictors of mental health service use among immigrant students. This scoping review aims to identify barriers and facilitators to mental health service use among immigrant students and categorize them for context-specific adaptation. Therefore, through this review, gaps in knowledge regarding the mental health of immigrant students and their use of mental health services will be identified, with the goal of making recommendations for improving mental health service provision and delivery to international students.

## Methods

### Protocol design

The full methodology (the protocol) of this review has been published [36].

### Stage 1: Identification of the research question and the objectives

The purpose of this Scoping Review is to provide a systematic map of research in this area and to identify existing gaps in what is known about the barriers and facilitators associated with access to and use of mental health services among immigrant students in high income countries.

Our scoping review was guided by the following research question: What are the factors that hinder (barriers) or help (facilitators) immigrant students in high-income countries access mental health services? To formulate this question, we utilized Cooke, Smith & Booth’s SPIDER tool, which aligned with our research objectives [37]. Our sample (S) consisted of immigrant students, and the phenomenon of interest (P of I) was the barriers and facilitators they encounter in accessing mental health services. We included all study designs (D), such as questionnaires, surveys, interviews, focus groups, case studies, or observational studies. We evaluated and assessed (E) students’ experiences in accessing care, including positive factors that facilitate access and negative factors that serve as barriers. Finally, we considered all types of research (R), including quantitative, qualitative, or mixed methods.

### Stage 2: Identifying relevant studies

#### Data sources

To ensure methodological transparency and obtain high-quality results and synthesized evidence, we developed and implemented a comprehensive research strategy, using the PRISMA-ScR checklist (S1 File) as a guide [38]. The scoping review was conducted following the guidelines for scoping reviews outlined by The Joanna Briggs Institute (JBI), based on the earlier work of Arksey and O’Malley [39] and improved by O’Brien, Colquhoun, Levac and colleagues [40].

An information specialist (K.F.) conducted a literature search in multiple databases, including MEDLINE(R) ALL (OvidSP), Embase (OvidSP), CINAHL (EBSCOHost), Education Source (EBSCOHost), and Web of Science, from the inception of the databases to January 27th, 2022. We used a combination of subject headings and keywords related to the concepts of "international or immigrant students," "mental health," and "access or use of health or mental health services." From these keywords, we developed MeSH terms for database searches. We searched for articles in these databases from their creation. An example of the final search strategy for MEDLINE is presented in S2 File. Search Terms.

We used the bibliographic software Zotero to store, organize, and manage all references [41] and Covidence (Veritas Health Information, Melbourne, Australia) to manage the title/abstract and full-text screening phases [42].

### Eligibility criteria

Studies were excluded if they were not conducted in high-income countries because they are the major destinations for immigrant and international students [43, 44]. Using the inclusion and exclusion criteria specified below, two independent reviewers screened each title and abstract on Covidence. We only included studies involving adolescents and older individuals in high-income countries. The primary outcomes we focused on were the facilitators and barriers to accessing mental health care or services.

#### Inclusion criteria

- Articles addressing access to and use of mental health care
- Articles about immigrant/international students
- Participants in the study are adolescents or older
- Original language is English
- High-income countries

#### Exclusion criteria

- Do not address access to and use of mental health care
- Not about immigrant’s/international’s students
- Studies involving on children (younger than adolescents)
- Language other than English
- Any commentaries, editorials, or opinion pieces
- Low-income countries

### Stage 3: Study selection

To ensure consistency among reviewers, the first two authors (C.D. and O.O.) screened a sample of 20 publications and discussed the results. They revised the screening and data extraction manual accordingly before commencing the screening process for this review. Working in pairs, C.D. and O.O. sequentially evaluated the titles, abstracts, and full text of all publications identified by the search for potentially relevant publications. In cases where there were disagreements on study selection and data extraction, consensus was reached through discussion.

**Fig 1:**
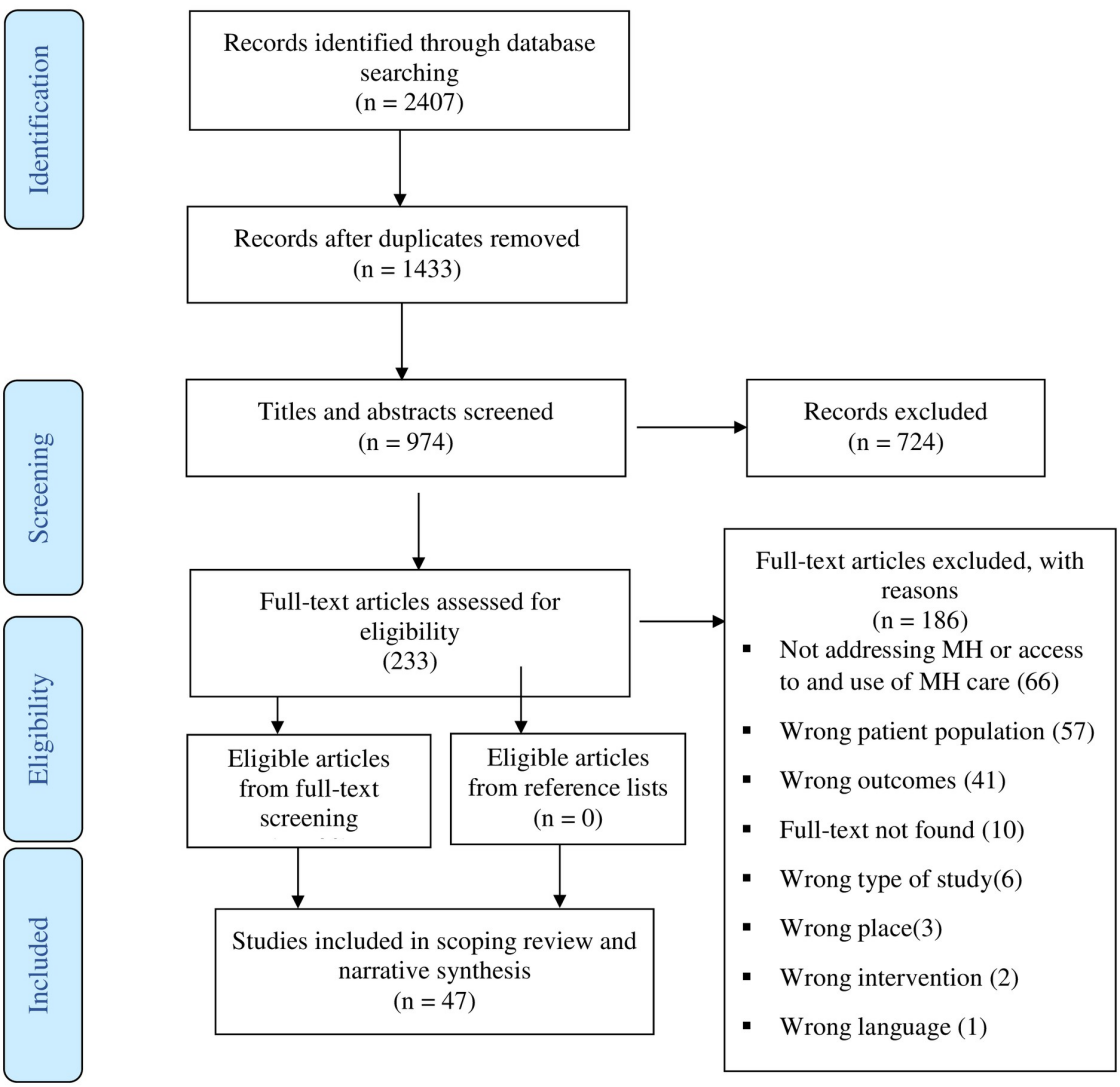
PRISMA flow chart.

Initially, our database search yielded 2407 abstracts published in English. After using Covidence [42] to eliminate duplicates, we were left with 974 articles to review. C.D. and O.O. reviewed the abstracts and excluded studies that did not meet the inclusion criteria. For instance, studies were excluded if the target population was clearly not immigrant students, the host country was not a high-income country, or the topic did not pertain to mental health or barriers and facilitators to mental health service utilization. This process resulted in 233 potentially relevant studies, for which full texts were reviewed. Ultimately, 47 studies met the inclusion criteria and were included in this review. Fig 1 presents the PRISMA flow chart for the selection of the included studies.

### Stage 4: Data extraction and charting the data

Each of the 47 studies included in this review was extracted and presented using an extraction template developed based on the Joanna Briggs Institute (JBI) Evidence Synthesis Manual [45]. We developed an Excel spreadsheet to record key information from the studies to be analyzed such as authors, references and relevant results or conclusions. We utilized a narrative review approach, which involved summarizing the key characteristics of each article, such as the country of origin, study type and nature, participant age group, date of publication, results, and more. The final version of the template included the following essential information: author(s); year of publication; study location; title; goals/objectives; participant demographics (age, gender, background, education level); methodology/methods (measures and tools employed); main findings related to the research question; barriers; facilitators; and recommendations.

### Methodological quality appraisal

Appraisal for quality assessment or risk of bias was not performed because this is a scoping review. This is consistent with the Joanna Briggs Institute Manual [45].

### Data summary and synthesis

Using Microsoft Excel, the data were organized in a spreadsheet and summarized quantitatively. Frequencies were calculated for the following variables: Year of publication, study setting, study country, study design, age group and gender of international students, country of origin of international students, barriers, and facilitators of utilization of mental health services and recommendations. Gaps in the literature were also identified. [Characteristics of included studies are detailed in the S3 File].

## Results

### Study characteristics

A total of forty-seven articles were included in this review on mental health service use and barriers and facilitators to service use among immigrant students. The characteristics of each of the qualitative, quantitative, and mixed methods studies are detailed in S3 File.

### Year and location of studies

The studies were all published between 1982 and 2022 and the majority (n = 40) were published between 2010 to 2022. All studies were conducted in a high-income country according to the World Bank classification [46]: United States (n = 36), Australia (n = 5), Canada (n = 2), Japan (n = 2), United Kingdom (n = 1) and New-Zealand (n = 1).

### Methodologies employed

Out of the total number of studies (47), 36 used quantitative methods while 8 were qualitative, and the remaining 3 were mixed methods. The quantitative studies involved the use of questionnaires/surveys to collect data, with 36 of them utilizing various measures commonly employed in the mental health field. Among these, four studies utilized secondary data, with the largest dataset obtained from the American College Health Association-National College Health Assessment (ACHA-NCHA) [47]. On the other hand, the qualitative studies [8] used Consensual Qualitative Research (CQR) and interviews in 7 of them. The mixed methods studies [3] employed a combination of questionnaires/surveys for the quantitative component and open-ended questions to gather qualitative data.

### Sample and participant characteristics

#### Sample size

The studies had varying numbers of participants, ranging from 2 to 10,730. The 36 quantitative studies had larger participant numbers, with a range of 45 to 10,730 individuals. In contrast, the qualitative studies had fewer participants, ranging from 2 to 33. The mixed studies had 11, 177, and 197 participants, respectively [48–50]. A significant number of participants in several studies were immigrant/international students. However, in at least 10 studies [47, 51–59], the representation of immigrant students in the total sample was small due to factors such as the study’s aim, setting, or ability to recruit immigrant students.

#### Age of participants

Data on the age of participants varied widely. Several studies gave an age range in years from 14 to 59 years, while others gave the average age of participants (16.6 years—28.9 years). However, three studies did not specify either the mean age or age range of participants [47, 60, 61]. But, since the sample of participants targeted was university students, it is very likely that they were adolescents or younger adults.

#### Gender of participants

Of the total studies (n = 45), most had both male and female participants, with females being predominant. However, Jones (2013) and Galligan (2017) studies had only female or male participants, respectively. Moreover, four articles referred to transgender and other gender identities to define gender among the participants [47, 57, 59, 62], in addition to men and women.

#### Origins of participants

Most studies included only immigrant student participants (n = 31) while others studied international students in comparison to domestic students (n = 17).

The majority of student participants in the studies were from Asia. Forty-six studies explicitly stated that at least one Asian country, such as China, Korea, Thailand, India, Vietnam, or Saudi Arabia, was the country of origin for the participating immigrant students. Only one study did not clearly specify the origin of its participants [63]. Among these studies, eight focused solely on Chinese immigrant students [24, 61, 64–68], two were exclusively on Korean students [69, 70], one on Saudi Arabian students [50], and one on Thai students [71].

Additionally, six studies mentioned African-origin student participants [22, 52, 62, 72, 73], and three studies included African Americans [51, 53, 62]. On the other hand, some studies referred to "other" backgrounds without specifying which ones precisely [51, 53, 60], and a study by Zhou, Zhou & Sun [2022] mentioned a range of nationalities from St. Vincent and the Grenadines to China. Only Meyer’s study in 2010 included Oceania as a student origin [52].

### Settings and target groups

Most of the included studies (n = 40) were conducted among university students and very often a mixture of undergraduate and graduate students. Some studies focused only on undergraduate students [56, 74, 75]. However, around 6 studies did not actually specify the degree or academic level of the students included. Five studies were conducted among college students [59, 73, 76–78] while only Arora and Algios’ study focused on high school students [78, 79].

### Problems encountered

Although all the studies in this review addressed in some way at least one challenge or problem encountered by immigrant students in their host country, most of the participants in these studies were not selected based on their specific mental health problem. The international students in the included studies in this review encountered several issues affecting their mental health: psychological issues (n≥35); socio-cultural stress (n≥26) and language issues (n≥5); personal and relationship issues (n≥8); educational challenges (n≥6); discrimination/racism/stigma (n≥8) and practical challenges (n≥4).

Immigrant students were primarily affected by psychological issues, such as depression (n = 18) and anxiety (n = 12), according to research. Additionally, socio-cultural challenges like acculturative stress are common among these students. The length of stay [51, 52, 68, 80] and English language proficiency [61, 66, 71, 81] have a significant impact on their level of acculturation, and those who struggle with these two factors are more likely to experience acculturative stress, which can exacerbate mental health problems [27, 82, 83]. The review also identified several personal/relationship [24, 50, 51, 53, 60, 70, 81, 84] and academic [47, 49, 56, 60, 62, 78] issues faced by immigrant students.

The literature suggests that immigrant students utilize mental health services less frequently than domestic students. Instead, they tend to seek help from informal sources, such as friends, relatives, partners, or religious leaders, when they encounter stress or problems. When these problems become more severe or deteriorate [49], they turn to formal sources of help, such as counselling, psychotherapy, or other mental health services provided by mental health professionals. Counselling is the most commonly used service by immigrant students, which is frequently available at their school and exposes them to therapeutic interventions or psychotherapy.

### Barriers

#### Barriers to mental health service utilization

The majority of the studies included in this review (n = 45) reported one or more barriers to accessing mental health services. These barriers were classified according to Wang et al.’s categorization from their 2019 study on Asian immigrant parents’ perception of barriers to school-based mental health services for adolescents. Wang et al.’s study relied on previous research on barriers to mental health services among Asians and Asian Americans and aimed to categorize the barriers to adolescents’ utilization of school-based mental health services. The authors suggested that these barriers are present among other culturally and linguistically diverse groups, such as Latinos and African Americans [85]. Therefore, we organized the barriers into five major categories: attitudinal, cultural, knowledge, structural/practical/logistical, and relational as shown in Table 1.

**Table 1:** An overview of key barriers and facilitators to mental health service use among immigrant students in high-incSome countries.

	Average frequency (number of studies)
**BARRIERS**	
**Attitudinal**	
Stigma, stereotypes, discrimination, doubt and fear	31
Masculinity/being a man	10
Lack of trust in professionals and the system	4
**Cultural**	
Being non-western or Asian	8
Cultural adherence (collectivism)	5
Lack of culturally appropriate services	3
**Knowledge**	
Lack of knowledge or information	10
Unperceived need	4
Doubt about usefulness of services	4
**Structural or Practical**	
Time	8
Cost	6
No previous contact	2
Doubt about confidentiality	3
**Social and relational**	
Functional relationships with counselors	1
Being in a group with fellow Asians	1
**FACILITATORS**	
**Attitudinal**	
Being female	7
Confidence in professionals and the system	4
Open-mindedness and high tolerance	3
Undergraduate students	2
**Cultural**	
Highly acculturated	6
Western or non-Asian origin	1
**Knowledge**	
Good mental health knowledge and literacy	5
Good English language skills and fluency	4
**Structural or Practical**	
Previous contact with services	4
Confidentiality	2
**Social and relational**	
Have good social connectivity	4
Have conflicts or relationship issues with counselors/teachers	2

#### Attitudinal barriers

These barriers pertain to the attitudes of immigrant students towards mental illness and mental health services in their host countries. These attitudes may also be influenced by their immediate environment, such as family and peers, or non-immediate environment, such as their cultural community or fellow citizens, which can affect their utilization of mental health services. Attitudinal barriers are characterized by prominent themes including stigma, stereotypes, discrimination, doubt, and fear (n = 31). These barriers are often associated with culture. For example, Lee’s 2014 study on the barriers faced by Asian international students when joining counseling groups highlights that they may be concerned about how others perceive them, particularly when seeking mental health assistance is stigmatized in their home culture [34].

Additionally, in quantitative studies conducted in United States, students were uncomfortable self-disclosing in the presence of a student from the same country of origin as they are, especially if their issues cannot be accepted by their ethnic community (e.g., sexual orientation). They would be more comfortable sharing their information with students from other countries than their own for fear of being stigmatized [34]. Also, according to Nguyen et al. in 2019, even for informal help, there would be a stigma, shame, and fear of sharing with others among international students. This is because they may not want to worry their family and loved ones, as they are often seen as the pride of their parents, or even their entire family. This makes it more difficult to exchange and share such problems with those back home [81]. Furthermore, certain parents of Asian immigrant students may experience shame with regards to their child’s use of mental health services due to the strong cultural pressure and collectivism that is often experienced by Asian nationals, even outside of their home country [79].

In several studies, it was found that masculinity or being male could be a barrier to mental health service utilization for many immigrant students, especially Asian students (n = 10). According to Galligan in 2017, norms of masculinity would be influenced by cultural norms and would advocate for the desire to manage oneself such as the desire to be seen as tough or strong, the feeling of not being able to cry and not having to express one’s emotions, and the desire not to be seen as weak [86]. Thus, the strong adherence to masculinity among men in Asian communities often leads to their reluctance in seeking mental health services when faced with mental health issues [49, 50, 52, 56, 63, 68, 74, 81, 86]. For instance, Alajlan’s study revealed that men were more likely to feel stigmatized by counseling and had lower tolerance scores compared to women [50]. Furthermore, being a man from an Asian community with a strong adherence to Asian culture could act as an additional barrier to accessing mental health services [50, 56, 81, 86].

Also, lack of trust in mental health services and professionals in the host country could be another barrier to using such services [54, 64, 71, 73, 76, 80, 86, 87]. These studies show that international students are skeptical and have some reservations about using mental health care. Tang et al.’s 2012 study reported that some students were not open to their new host society or were even culturally suspicious [75]. According to the authors, these students would not believe in the effectiveness of the services, the legitimacy of the professionals, and their ability to understand them to help them effectively. In addition, Wong’s 2014 American study shows that Asian international students who strongly adhered to emotional self-control and humility tended to have negative attitudes toward seeking professional psychological help [35]. According to the author, those who strongly adhere to the cultural value of humility may be uncomfortable asserting their needs and thus may have more negative attitudes due to the perception that psychotherapy encourages a focus on the self, which would be contrary to their value [35].

#### Cultural barriers

These are all barriers related to the original culture of the immigrant students and its interaction with the culture of the host country. The mismatch between the cultural aspects of the two countries (home and host) [71] can create a culture shock for these students who must somehow adapt to it in order to succeed. While most of the cultural barriers identified in this review are specific to the students themselves, some also relate to the mental health professionals they encounter.

Several studies have reported that being a non-Western student, and particularly Asian, can be a barrier to seeking mental health care (n = 8). Dadfar and Friedlander’s 1982 study reported that being a non-western student (African and Asian) could be a barrier to mental health service use [72]. Authors have indicated that strong ethnic identity, adherence to traditional values or cultural pressure on immigrant students, particularly Asians, may inhibit their openness and willingness to use mental health services (n = 8). For example, Santiago’s 2006 study indicated that the Asian identity of international students, particularly those from the Far East (e.g., China, Japan, both Koreas), who strongly adhere to traditional Asian values, would be a barrier [88]. Xie’s 2008 study of Chinese international students in the U.S. shows that many participants had an ambivalent attitude toward counseling; on an intellectual level, they thought that counseling could be helpful, but on a more personal level, they doubted the effectiveness because their cultural background would not be understood during such counseling [64]. However, Li et al.’s 2013 study noted that Asian international students tended to have more favourable attitudes towards mental health services than towards traditional forms of help specific to their culture [49].

Several studies indicate that the lack of culturally appropriate mental health services can also hinder immigrant students’ use of such services in their host countries [50, 52, 58, 60, 84]. These studies reveal that mental health care professionals’ lack of cultural competence which can result in immigrant students feeling misunderstood in terms of their culture. For instance, a qualitative investigation by Rogers-Sirin et al. (2015) on immigrant perceptions of therapist cultural competence found that therapists’ cultural competence significantly impacts their impressions of therapy, which can lead them to avoid further consultation [73]. However, Meyer’s (2009) research on international students from various backgrounds in the Boston School of Architecture in the US revealed that strong acculturation was unexpectedly a barrier [52]. The author suggests that this may be because developing English proficiency increases exposure to stigma within the host culture while reducing psychological distress as the individual becomes more culturally competent, which could reduce help-seeking attitudes and use of psychological services.

#### Barriers related to knowledge

This refers to lack of knowledge/low level of information about mental health services (or the host country system itself) and the identification of mental health symptoms/illnesses. Thus, lack of perception or doubt about the usefulness of mental health services and non-familiarity with the system are factors that may deter immigrant students from using mental health services in their host country.

About ten studies cite low knowledge and lack of information as a barrier to service utilization by immigrant students [22, 47, 51, 60, 63, 64, 78, 79, 86, 89]. The 2019 study by Clough et al. that examined mental health knowledge and help-seeking attitudes between domestic and international students in Australia showed that low levels of mental health knowledge were related to less favorable help-seeking attitudes. It found that mental health literacy was also lower among males, students who had recently arrived in Australia, and students who had never had contact with mental health services [63].

Some authors have suggested that students who lack knowledge about mental health often do not perceive the need for mental health services [57, 59, 64, 78, 86]. Zhou, Zhou & Sun find in their study in 2021 that there’s a significant disparity between students (especially among international students) in need of services and those who receive services, which indicates that not all those who screened positive were aware of their need for services. So, there was low perceived need of services. This can be associated with doubts about the actual usefulness of mental health services and thus may be a barrier to utilization of these services [54, 63, 76, 78, 79, 86]. Xie’s 2008 research revealed that although most participants in the study were aware of the existence of mental health services and demonstrated some level of understanding of psychological counselling concepts, many reported being unfamiliar with the counselling process and procedures [64]. In addition, Rhee’s research work showed that language difficulties such as lack of fluency in English are barriers to utilizing mental health services. The author found that some Thai students in the U.S. preferred to see a Thai counsellor as they believed the Thai counsellor would have a better understanding of their situation, especially since certain Thai words cannot be translated into English [71].

#### Structural, practical/logistical barriers

Structural, practical, and logistical barriers are often not dependent on the students themselves, but rather on host country factors. For the most part, these are factors that impede access to mental health care.

Time or time-related factors have been mentioned in several studies (n = 8) [24, 50, 63–65, 78, 86, 89]. The long-time due to long waiting lists could demotivate immigrant students to use mental health services. The 2020 study by Lian et al. noted, for example, that full-time employed students, very often older and graduate students, were less likely to use mental health services often due to lack of time [24].

Cost can be a barrier to access and use of mental health services [50, 64, 71, 86, 87, 89, 90]. Rhee’s 2018 study reveals, for example, that the Thai international students in the study seemed to prefer using social support over services with professionals because of the associated expenses. Since they typically do not have access to student loans like American students and use personal savings to pay for their education, they are very often on a tight budget and therefore cannot afford additional expenses while in school [71].

The lack of prior experience with mental health services [54, 63, 72] and concerns about the confidentiality of counseling sessions [64, 78, 86] are additional barriers to immigrant students accessing care in their host countries. Clough et al. [2019] found that international students who had never used mental health services had less knowledge about mental health, making it difficult for them to seek services for the first time [63]. In another study by Cha et al. [2019], students without legal documentation expressed greater concerns about the confidentiality of counseling sessions, which may discourage them from seeking services [78].

#### Social/relational barriers

These barriers are due to the relationships that immigrant students have in their host country or in their country of origin. These relationships can be with members of their respective communities or people within their institution such as their advisors. Studies have demonstrated that Asian students, who had strong cultural adherence and thus collectivism remained strong even outside their home country [34, 49, 55, 66, 67, 70, 84, 91] preferred not to use services especially when there were other fellow citizens in the sessions. For example, Lee’s 2014 study that examined Asian international students’ barriers to participating in counseling groups showed that they were uncomfortable and did not like participating in counseling groups with other Asian students [34]. They preferred to be in groups with other domestic or international students so that they would not be stigmatized and lose face in front of their community [34].

Also, Hyun’s 2007 study found that international students who had a good functional relationship with their advisors (academic/academic advisor, supervisor, professional career advisor, or any other type of advisor) were less likely to have had a stress-related problem or an emotional problem. They were then less likely to use mental health services [51]. The study of Lee & al. in 2017 also found that international Asian students in New-Zealand who have a good relationship with their parents were less likely to use the services [87].

### Facilitators

#### Facilitators to mental health service utilization

Facilitators are factors that makes it easier or more likely that students will seek mental health care. Thus, any positive experience that immigrant or international students have would be considered a facilitator. In contrast to barriers, we identified fewer facilitators and some studies reported none [35, 55, 56, 58, 59, 68, 70, 76, 78, 89, 92]. As with barriers, we also drew on the categorization by Wang et al. to group facilitators (attitudinal, cultural, knowledge-related, structural or practical/logistical and relational) to mental health service use [85]. Similarly, for each of these categories, similar data were grouped into themes developed from the terminology used in the literature reviewed.

#### Attitudinal facilitators

Attitudinal facilitators are factors related to the attitudes or behaviours of immigrant students that are thought to have a positive impact on mental health care utilization. Several studies have reported that women are more likely to seek help [49, 50, 52, 54, 60, 74, 77, 81]. Although these authors offer little explanation for this, it is believed that unlike men who have a high degree of masculinity (considered a barrier), women are more tolerant and willing to use mental health services [50]. For example, international female students used counseling services more often than males in most of these studies [54, 60, 74, 77].

Having confidence in mental health professionals and the system may also facilitate the use of mental health services by immigrant students [75, 79, 80, 84]. Indeed, students who have confidence in professionals, especially after an initial contact [64], would be more likely to return for further consultation when necessary. In terms of the system, it is those who are confident of safety and privacy who would be more confident in using mental health services [79].

Openness and high tolerance, which is highly dependent on the home culture, but also on the host culture, could facilitate students’ use of mental health care [50, 64, 80]. Openness depends largely on the students’ own state of mind and this is often induced by the level of acculturation or time spent in the host country [80] or perhaps related to several other factors [64]. Indeed, Li et al.’s 2013 study explains that many Asian countries such as China are characterized by the collectivist culture, which is characterized by a group-oriented social identity (the family and kinship groups). Thus, the success or failure of an individual is not only a personal matter but concerns the whole family and the community. This group pressure may cause individuals to hide embarrassing events in their lives to protect their family honour and to do their best to resolve them themselves. Hence, they use emotional self-control and resilience, which are considered virtues, especially during difficult life events [49]. These cultural norms and values explain why Asian international students often view counseling as the last resort for solving life problems, but, those who are more open and tolerant are more likely to consider and utilize services [50, 64, 80].

Several studies revealed that undergraduates had more mental health consultations than graduate students [24, 49]. Thus, being an undergraduate could play a facilitating role in mental health service utilization. In Lian et al.’s 2020 study, Chinese graduate students had lower levels of help-seeking intentions compared to undergraduate students [24]. However, the author noted that the undergraduate student population was a small proportion. Thus, interpretation should be made with care [24]. In addition, Hyun’s 2007 study found that Asian graduate students who were experiencing financial difficulties or difficulties completing their course or semester were more likely to seek counseling [51]. Thus, international graduate students tend to seek counseling for problems that are less psychological or emotional. Also, according to Nguyen et al. in 2019, age may be a significant predictor of help-seeking behaviors among students and older international students who are typically graduated, less likely to cope with their emotional problems that would cause them to seek help [81]. In contrast, the authors stated that the correlation between age and willingness to self-help among international students may be difficult to explain due to the dynamics of cultural difference among the students in the study [81].

#### Cultural facilitators

Cultural facilitators are factors related to the culture of immigrant students that play a positive role in their use of mental health services in their host country. Strong acculturation, which is the process of breaking away from one’s original culture and adopting the culture of one’s host country for easy integration [83], seems to be a facilitator of mental health service utilization. Studies have found that the stronger the acculturation of immigrant students, the less adherent they were to their native culture, the more open they were to mental health care and the easier it was for them to use mental health services [34, 51, 66, 67, 71, 80, 81, 84]. A cross-sectional study conducted by Li, Marbley, Bradley & Lan in United-States in 2016 demonstrated that acculturation, ethnic identity, and English proficiency were statistically significant predictors for Attitudes Toward Seeking Professional Counseling Services among Chinese International students. Similarly, the quantitative study by Zhang and Dixon in 2003 showed that there was a significant positive relationship between Asian international students’ level of acculturation, tolerance, and confidence in mental health practitioners. Thus, students who were more acculturated were also more tolerant of mental illness and had greater confidence in mental health professionals and thus, more willingness to consult them [80]. Furthermore, Nguyen et al. found that acculturation stress was a significant predictor of help-seeking and willingness to seek formal help for international students [81]. Thus, integrated acculturation strategies tend to be associated with the most positive attitude toward mental health services as in Chauv’s cross-sectional study in the United States in 2020 that predicted the use of mental health professionals [81].

The backgrounds and origins of individuals may act as a catalyst in certain instances. Studies have suggested that immigrant students from Western countries tend to be more inclined to utilize services compared to those from non-Western backgrounds [72, 74]. Santiago’s research has also indicated that South Asian students are more prone to using mental health services compared to their peers from other Asian countries, such as those in the Far East [88]. It seems that individuals who do not strongly adhere to traditional Asian values are more open to integrating with the Western culture and utilizing mental health services [84].

#### Knowledge-related facilitators

These are factors related to students’ level of knowledge that would have an impact on immigrant students’ use of mental health services. Having good knowledge about mental health [51, 63, 64] and being aware of existing services [51] has the potential to facilitate students’ use of mental health services. Australian studies by Clough et al. in 2019 and 2020 on mental health literacy show that immigrant students with good mental health literacy are more likely to use services [22, 63]. The good mental health literacy of international and domestic students could enable them to identify early signs and symptoms of mental disorders and thus seek help when needed [63]. In addition, immigrant students who had experience with mental health and psycho-emotional challenges were more likely to use services [64]. The severity of the disorder could also play a positive role in their use of mental health services [24, 81, 86]. Moreover, research indicates that immigrant students who have had experienced with mental health and psycho-emotional difficulties are more inclined to seek mental health services [64]. Furthermore, the severity of the mental health problem may have a beneficial effect on their utilization of mental health services [24, 81, 86].

Furthermore, possessing a strong command of the English language and being fluent in it can aid in the utilization of mental health services by immigrant students [61, 66, 81, 84]. Being proficient in English enhances communication and boosts the confidence of immigrant students, enabling them to seek assistance and utilize services with greater ease. Additionally, immigrant students’ proficiency in English is linked to their level of acculturation, which also acts as a facilitator [84]. Mesidor and Sly [2014] also suggested that having a sense of control over one’s behavior could motivate immigrant students to seek mental health care [53].

#### Structural or practical/logistical facilitators

These are facilitators related to the accessibility of mental health services. Studies have reported that previous contact with services play a facilitating role in the use of mental health services [49, 64, 72, 91]. In these studies, students who have used mental health services in the past are more likely to use them again, especially if their first experience was positive [64]. In addition, Arora and Algios’ study showed that immigrant students are more willing to use mental health services when they feel confident that their privacy is well protected [79].

#### Social/relational facilitators

The social and relational factors that may prompt immigrant students to use mental health services are numerous. Certain studies indicate that conflicts or relationship issues with their counselors or professors could facilitate the utilization of mental health services among immigrant students [64, 86]. Since these students frequently have a restricted social network in their host country, their professors or counselors hold significant importance to them. As a result, conflicts with such figures could have a more profound psychological impact and motivate them to seek help. Lian et al. reported that American professors were among the preferred sources of aid for Chinese international students, albeit not the primary one [24]. Additionally, a strained relationship with an advisor might be linked to greater mental health symptoms [24, 51] that could result in seeking assistance. BongJoo et al.’s research in 2014 revealed that international students are more likely to seek referrals from authority figures rather than seeking help independently [60].

Furthermore, possessing a strong social support system is considered a crucial factor in promoting the utilization of mental health services [24, 81, 84]. Having a robust support network means having individuals with whom one can share their difficulties and seek assistance [24, 87]. For instance, in Lian et al.’s study in 2020, some immigrant students reported using mental health services after receiving advice or information from a friend, relative, or partner [24]. Interestingly, some studies have identified unexpected and counterintuitive facilitators, such as stress arising from racism [88], feelings of isolation [86], or being in the same group as other international students [34].

## Discussion

The mental health of immigrant students is a complex and increasingly researched topic. This review focuses on high-income countries, such as the United States, the United Kingdom, Canada, China, and Australia [46]. These countries alone hosted more than half of the world’s international students in 2020, according to Project Atlas [43, 44]. Notably, the majority of studies (N = 36) were conducted and published in the US, which hosted over one million international students (20%) in 2020 [43]. In contrast, the limited number of studies in other high-income countries, such as Australia (N = 5), Canada (N = 2), Japan (N = 2), the UK (N = 1), and New Zealand (N = 1), raises questions. It is difficult to explain why few, or no studies have been conducted in countries with significant numbers of immigrant students, such as China, Germany, or France. For instance, the French Ministry of Higher Education and Research reported that international students made up 11% of the student population in 2022 [93], yet we could not find any studies on their mental health. It is worth noting that restricting the review to English-language articles may have contributed to the lack of studies in other languages, such as French, German, or Mandarin.

In addition, in China the total cost of mental disorders in 2013 accounted for more than 15% of total health expenditures in China, and 1.1% of GDP [94]. In the UK in 2013, mental health problems cost the British economy nearly £100 billion, or 4.5% of GDP [95]. In Australia, the Australian government spent nearly A$3.6 billion on mental health services in 2018–19 [96]. More recently in Canada, the federal government invested nearly $994.6 million Canadian dollars in 2021 on mental health care and essential social services to enable good mental health for more people in the country [97]. These figures suggest that these countries have some commitment to the mental health of populations in general. While it is difficult to know whether international students are among the populations targeted by these government investments, we do know that in Canada, for example, as temporary immigrants, they are not often included in government-funded programs as are other permanent resident immigrants [33]. Yet they contribute significantly to the social and economic development of these countries. For example, international students contributed nearly $29 billion to the Australian economy in 2022 [98]. In Canada, they contributed nearly $21.6 billion to the country’s GDP in 2018 [99]. According to London Economics, international first-year students enrolled in UK higher education institutions had an estimated impact on the economy of £25.9 billion in the 2018–2019 academic year [100].

Predictably, most immigrant students in this review were of Asian, particularly Chinese, origin. Indeed, Asian countries send the most nationals to study abroad. According to Project Atlas [43], in each of the top five countries hosting the most international students in the world, the first country of origin of those students was an Asian country. China, for example, is the top country sending the most students to study in the U.S., the U.K. and Australia [43]. And the latter is also among the top five countries that host the most international students with the majority of international students coming from neighboring Asian countries such as South Korea, Thailand, Pakistan and India [43].

Results show that immigrant students use mental health services less than domestic students. Those who experience difficulties first try to manage them themselves [35] or seek help from informal sources [62, 89] such as friends, relatives and intimate partners [24, 81]. It is when they face more serious problems [49] that they seek formal help such as counselling [22, 34, 47, 49–51, 53, 60, 64–66, 68–71, 74, 76–78, 84]; psychotherapy [47, 73, 75]; or general psychological help from mental health professionals (n = 14) [24, 52, 59, 61, 63, 64, 69, 70, 78, 79, 81, 90–92]. Consistent with the literature, Kim et al. showed in 1999 that culture plays an important role in help seeking [101]. According to the authors, in Asian culture, people express emotions less than in Western and non-Asian cultures [101]. This may be the reason why Asian students tend to be more self-controlled than help-seeking, and may also lack trust in mental health services and professionals in the host country [64, 71, 80, 86].

Furthermore, in this review we identified important reasons for underutilization of mental health services among international students in high income countries such as stigma, stereotypes, discrimination, doubt and fear; masculinity or being male [49, 50, 52, 56, 63, 68, 74, 81, 86] and cultural barriers [6, 9, 25, 26]; knowledge barriers [22, 47, 51, 60, 63, 64, 78, 79, 86, 89], practical barriers such as time [24, 50, 63–65, 78, 86, 89], cost [50, 64, 71, 86, 87, 89, 90]; confidentiality [64, 78, 86] and social/relational barriers [34, 49, 55, 66, 67, 70, 84, 91], and others may inhibit the use of mental health services. These data are consistent with the barriers faced by immigrants in general. In Amelia Derr’s 2016 systematic review, cultural (stigma, norms, and attitudes), structural (cost, transportation, lack of insurance, and discrimination), language, and knowledge barriers were the main barriers encountered by immigrants in the US [102].

This review also identified key facilitators to mental health service utilization among immigrant students such as being female [49, 50, 52, 54, 60, 74, 77, 81], trusting mental health professionals [75, 79, 80, 84], being open-minded [50, 64, 80], having good knowledge [2, 6, 10, 11, 24], having good social connectedness [24, 81, 84, 87], acculturation [34, 51, 66, 67, 71, 80, 81, 84], previous contact with services [49, 64, 72, 91], having a good level of English and fluency in English [61, 66, 81, 84]. These data are also consistent with facilitators of mental health utilization in the general immigrant population in high income countries [12, 102].

### Recommendations to improve mental health of immigrant students

The methods of accessing and delivering mental health care to immigrant students require many improvements. Based on this review, we identified key factors that might enable immigrant students to utilize mental health services in their host countries. First, education and awareness [22, 24, 35, 49–52, 55, 61–65, 67, 68, 70, 71, 73–75, 77, 79, 84, 87, 88, 103]. Thus, there is a need for concerted effort by host countries to provide adequate and on ongoing accessible information to immigrant students in their countries on the importance of accessing mental health care. This includes raising awareness about mental health issues [47], educating them about the benefits of counselling services [71], explaining the counselling process and the different ways to participate [35]. This can increase their familiarity with counselling services [49]. Second, collaboration [24, 49, 50, 62, 64, 68, 78, 79, 84, 86] through the involvement of peer mentors adopting therapeutic approaches that are appropriate to the diverse immigrant student population [49, 62, 64, 70, 75, 84, 89]. Some studies recommend the use of psychodynamic and more integrated approaches such as cognitive behavioural therapy (CBT) [64] with person-centred counselling [64, 75]. Mindfulness-based approaches that draw on Eastern philosophy (such as mindfulness and decentering) [75] have been suggested as potentially more appropriate and sensitive to the needs of people from Eastern cultures. Community-based intervention programs that gather stories from former clients and their families have also been suggested as a way to reduce the stigma associated with mental illness and mental health counselling [70]. Third, improvement in the accessibility of mental health services with the assurance of confidentiality of sessions [22, 34, 54, 61, 62, 65, 71, 79] would encourage immigrant students to access mental health services; Fourth, the need to adapt the services, so that services are culturally safe and appropriate for immigrant students [66, 90].

### Strengths and limitations of this scoping review

A strength of this review is that we followed a predefined protocol [36] that enabled a well-structured, rigorous, and systematic approach in arriving at results. Additionally, based on a set of predefined eligibility criteria studies were carefully selected and have yielded potentially useful information regarding the barriers and facilitators of mental health services among immigrant students in high income countries. However, there are several limitations in this review that need to be considered. First, the search strategy may have missed relevant articles. According to McDonald et al, the coverage of journal articles to be included in the review is influenced by the choice of database [104]. In this review, we used five databases, and some relevant studies might not have been in these databases especially if the keywords used in the search strategy had not been broad enough to encompass all published research on the topic. In addition, the selection of keywords in the search strategy as well as the databases was done with the help of a librarian specialized in the field. In addition, human errors could have been introduced in the different stages of the selection. For example, during the different sorts (especially in step 1: *sorting of the abstract and title*), relevant articles could have been eliminated because the keywords were not present in the title and abstract.

Second, another potential limitation of this review is the restriction placed on the language and type of articles included. By only including articles written in English, we may have overlooked relevant studies published in other languages. For instance, China, which is among the top five countries hosting the largest number of international students as of 2019 [43], primarily uses Mandarin. As a result, we may have missed out on pertinent articles written in Mandarin or other Chinese languages by restricting our search to English-only articles.

Furthermore, it may be difficult to generalize the results of these studies given the large cultural gap between the different host countries and countries of origin of immigrant students. Although, we have only considered high-income countries in this review to minimize the differences between them (economic, social, demographic, political, cultural, etc.) to better study them, these students themselves come from countries that often have very different socio-political-economic and cultural situations. For example, studies show that many immigrant students come from Asia, particularly China, choose the U.S. as their preferred destination for study. However, there is a great deal of cultural difference between these countries, especially with respect to mental health and the use of mental health services [49, 70, 84, 86, 88]. It is therefore difficult to generalize the results of these studies; interpretation should be made according to each context.

### Recommendations for future research

All the studies included in this review had a cross-sectional design, which precludes establishing any causal relationships between the factors (barriers and facilitators) and the use of mental health services. Therefore, future research should concentrate on longitudinal or intensive case studies that track immigrant students over time to determine how service use evolves after their arrival in the host country, as well as the underlying factors that affect help-seeking behaviors among immigrant students in high-income nations [102]. Additionally, a substantial number of the studies in this review were conducted among Asian students, there is a need for more studies to be conducted among other prominent student population groups such as Africans and Latinos in high income countries to have a holistic view of the barriers and facilitators of mental health use among immigrant students. Also, future research should not treat immigrant students as a monolithic group in research, given the considerable variabilities that exist among international students from different countries in terms of culture, social norms, experiences, and help-seeking behaviors.

## Conclusion

Immigrant and international students’ unique experiences and mental health needs are often unmet, as indicated by the numerous barriers identified in this study. To address these gaps, tailored approaches based on their life context and primary prevention methods such as education and mental health promotion are necessary. Intervention programs that integrate formal and informal services such as psychodynamic and CBT approaches can be effective.

## Supporting information

S1 File PRISMA-ScR checklist. (DOCX)

S2 File Search terms. (XLSX)

S3 File Characteristics of the studies. (DOCX)
