# Supplementary material for: Barriers and facilitators associated with the use of mental health services among immigrant students in high-income countries: A systematic scoping review
Source: PLoS One. 2023 Jun 29;18(6):e0287162. doi: 10.1371/journal.pone.0287162 (PMC10310021; doi:10.1371/journal.pone.0287162)
Supplement: S3 File — (DOCX) [file pone.0287162.s003.docx]

Additional file 2: Search Terms

| Ovid MEDLINE(R) ALL <1946 to January 26, 2022> | | |
| --- | --- | --- |
| **#** | **Search terms** | **Results** |
| 1 | Mental Health/ | 50297 |
| 2 | mental health*.ti,ab,kf. | 188803 |
| 3 | exp Mental Disorders/ | 1347077 |
| 4 | Depression/ | 136990 |
| 5 | (mental* adj2 (disorder* or disease* or ill* or condition*)).ti,ab,kf. | 107236 |
| 6 | (depress* or anxiet* or anxious or posttraumatic* or post-traumatic* or suicide* or suicidal* or (psycholog* adj3 (distress* or suffer* or condition* or disorder* or ill* or disease*))).ti,ab,kf. | 773375 |
| 7 | ((substance* or drug*) adj2 (consumption* or use* or using or abus*)).ti,ab,kf. | 240724 |
| 8 | or/1-7 | 2101915 |
| 9 | exp Health Services Accessibility/ | 120851 |
| 10 | (healthcare adj3 (access* or equit*)).ti,ab,kf. | 9076 |
| 11 | (health adj2 (service* or care) adj2 (seek* or use* or using or utiliz* or access* or equit*)).ti,ab,kf. | 53586 |
| 12 | Health card*.ti,ab,kf. | 714 |
| 13 | exp Insurance/ | 192582 |
| 14 | Insurance*.ti,ab,kf. | 101117 |
| 15 | (mental health adj2 (seek* or access* or use* or using or utiliz* or service* or care)).ti,ab,kf. | 41026 |
| 16 | ((use* or using or utiliz* or access* or seek*) adj3 (counsell* or psychotherap* or psycho-therap* or therap*)).ti,ab,kf. | 244134 |
| 17 | help-seeking behavior/ | 1077 |
| 18 | (help adj2 seek*).ti,ab,kf. | 12558 |
| 19 | or/9-18 | 678966 |
| 20 | exp Students/ | 149778 |
| 21 | student*.ti,ab,kf. | 320308 |
| 22 | or/20-21 | 356314 |
| 23 | exp "Emigrants and Immigrants"/ | 14394 |
| 24 | Emigration and Immigration/ | 25809 |
| 25 | Refugees/ | 11836 |
| 26 | (immigra* or emigra* or refugee* or asylum seeker*).ti,ab,kf. | 55222 |
| 27 | or/23-26 | 76279 |
| 28 | 22 and 27 | 2199 |
| 29 | ((Exchange* or International* or permanent resident*) adj3 student*).ti,ab,kf. | 1868 |
| 30 | ((Cultur* or linguisti*) adj2 divers* adj3 student*).ti,ab,kf. | 214 |
| 31 | or/28-30 | 4135 |
| 32 | 8 and 19 and 31 | 93 |
